# Adolescents' responses to online peer conflict: How self‐evaluation and ethnicity matter

**DOI:** 10.1002/icd.2067

**Published:** 2017-11-23

**Authors:** Sheida Novin, Marieke G.N. Bos, Claire E. Stevenson, Carolien Rieffe

**Affiliations:** ^1^ Utrecht University Utrecht The Netherlands; ^2^ Leiden University Leiden The Netherlands; ^3^ University of Amsterdam Amsterdam The Netherlands; ^4^ Dutch Foundation for the Deaf and Hard Hearing Child Amsterdam The Netherlands; ^5^ University College London London UK

**Keywords:** aggression, cyber bullying, emotion, self‐esteem, sense of coherence

## Abstract

For parents, online platforms where their children interact with others often feel like a “black box” in terms of what exactly is happening. In this study, we developed an ecologically valid online computer game in which a (computer‐generated) peer teammate tried to provoke frustration, in order to examine (a) adolescents' responses and (b) how indices of self‐evaluation (i.e., sense of coherence and self‐esteem) and demographic variables (i.e., gender and ethnicity) matter to these responses. Like gender, being a member of a minority or majority group may influence how provocations by peers are interpreted, influencing how one responds. Fifteen‐year‐old Dutch and Moroccan‐Dutch adolescents (N = 167) completed self‐reports and played the online computer game. The game indeed elicited frustration, with increased self‐reported anger. Moreover, expressions of displeasure were much more common during and after provocation than before provocation. Crucially, perceived self‐evaluation mattered; higher levels of sense of coherence but lower levels of self‐esteem (only in Moroccan‐Dutch group) contributed to fewer expressions of displeasure. Gender did not play a moderating role. Our findings provide initial insights into individual differences in adolescents' responses in an online peer‐conflict situation.

**Highlights:**

- We studied Dutch and Moroccan-Dutch adolescents' responses during online peer provocation and how self-evaluation and demographic variables matter.
- Provocation by the (computer-generated) peer teammate increased expressions of displeasure.
- More sense of coherence but less self-esteem was associated with fewer expressions of displeasure, but ethnicity moderated the effect with self-esteem.

## 1 INTRODUCTION

In the digital age we live in, children and adolescents have easy access to various social media websites and online communities, which create platforms for youngsters to interact with peers beyond the schoolyard. Although these interactions can be entertaining and valuable, the relatively anonymous nature of the interactions increases the probability that youngsters are targeted, provoked, or even harassed. Indeed, in a European study among children and teenagers, 11–23% reported feeling upset, uncomfortable, and bothered when using the Internet (Livingstone, Mascheroni, Ólafsson, & Haddon, 2014). Although children's and adolescents' responses to peer provocation have been studied using hypothetical scenarios (e.g., Dirks, Suor, Rusch, & Frazier, 2014; Novin, Banerjee, & Rieffe, 2012) and naturalistic or lab observations (Leary & Katz, 2005; Underwood & Bjornstad, 2001), much less is known about their responses when being provoked online. Obtaining insight into how youngsters respond to online peer provocation is important, given that certain responses can exacerbate the provocation leading to systematic cyber bullying (Willard, 2007) and/or psychological problems (Reijntjes, Kamphuis, Prinzie, & Telch, 2010). Our current study contributes to the field in at least two ways: (a) by examining adolescents' expressions of displeasure during online peer provocation using a newly developed ecologically valid computerized game and (b) by examining specific adolescent characteristics that might influence these expressions.

### 1.1 Responses to peer provocation

Conflicts in which peers provoke each other are largely unavoidable in adolescence, whether these occur between close friends, classmates, or strangers (e.g., Card & Hodges, 2008). Learning how to manage interpersonal conflict situations is a major developmental task during adolescence. Adolescence is a period that is characterized on the one hand, by the need to strive for independence and on the other hand, by an increased social sensitivity in which youngsters are preoccupied with how they appear to others (Blakemore & Mills, 2014). Moreover, due to the major physical, cognitive, and psychological changes, emotional experiences in adolescence are more frequent and intense than before or after this period (Heller & Casey, 2016). Taken together, these characteristics make dealing with peer provocations a challenging task.

Observational studies as well as studies using hypothetical scenarios show that youngsters vary in how they respond to peer provocations (e.g., Dirks, Suol et al., 2014; Dirks, Treat, & Weersing, 2007; Underwood & Bjornstad, 2001). Some have the tendency to neutralize the conflict situation, including responding positively or with humour, talking about something else, or trying to solve the problem. Others respond in ways that show one's displeasure, including verbally objecting, venting, or even using aggression. Studies show that when being provoked, expressions of displeasure towards the provoking peer could escalate the peer‐conflict situation (Murphy & Eisenberg, 2002).

Going beyond observational and hypothetical studies, there appears to be a lack of knowledge of how adolescents respond to online peer provocations despite the fact that the majority of western adolescents use the Internet for communication purposes on a daily basis (Livingstone et al., 2014). As such, it is likely that youngsters will be confronted with “trolls” (those who intentionally act provokingly, Griffiths, 2014) or with “grievers” (those who try to ruin the gaming experience by for example team killing; Adrian, 2010) at some point in time. In various self‐report studies, adolescents were asked what they would do if they were bullied online. Confronting the bully and retaliating were commonly reported (DiBasilio, 2008; Stacey, 2009), but within the group who actually reported to be victims, many would (pretend to) ignore it (61%; Dehue, Bolman, & Vollink, 2008). Although informative, these studies have two drawbacks.

First, self‐report information of how one responds to provocations do not necessarily correspond to actual responses in a real‐life situation (e.g., Robinson & Clore, 2002; Underwood & Bjornstad, 2001). In addition to possible response biases (social desirability) when using self‐reports, differences between self‐reports and actual responses are likely to be due to differences in what is accessible in one's memory at the moment of judgement (e.g., Fiske, 1992). Cognitive and behavioural responses to real‐time situations are episodic in nature, that is, situation specific and based on what one is feeling (Robinson & Clore, 2002). Reported responses to hypothetical situations, however, are semantic in nature, that is, decontextualized and based on general beliefs about emotions and emotional responses. Consequently, the use of an ecologically valid measure to study adolescents' responses to online peer provocation is essential. We newly developed such a measure in the current study.

Second, prior work focusing on responses to online peer provocation does not provide insight into which and how individual differences matter. Why are some adolescents likely to show their displeasure, whereas others are not? As discussed in detail below, in the current study, we focused on adolescents' self‐evaluation (i.e., sense of coherence and self‐esteem) and demographic characteristics (i.e., gender and ethnicity) to better understand individual differences in adolescents' expressions of displeasure when being provoked online by a peer.

### 1.2 Sense of coherence and self‐esteem

Sense of coherence (SOC) and self‐esteem can both be considered self‐evaluative constructs that reflect how one perceives oneself and one's capabilities. SOC refers to the evaluation of one's capability to respond appropriately and adaptively to difficult or challenging (social) situations. High levels of sense of coherence are derived from perceiving the world as comprehensible (situations are predictable and explicable), manageable (resources are available to meet the demands of the situation), and meaningful (situations are challenging and worthy to invest and engage in) (Antonovsky, 1979). Self‐esteem can be considered a more global construct referring to a person's evaluation of self‐worth, including feeling competent and confident (Harter, 2006).

Self‐evaluations such as self‐esteem and SOC are especially salient in adolescence, a period in which youngsters become increasingly preoccupied with the (dis)congruence between their ideal and real self (Harter, 2012). In their struggle to find “the real me”, opinions of significant others are very influential as social awareness dramatically increases during this life period. Acting in ways that are true to oneself is often on adolescents' mind and should influence how they react to various situations, including when being provoked in an online setting.

Prior work indeed shows that both adolescents' and adults' beliefs in their abilities (self‐efficacy) are related to how they respond to witnessing online provocations or bullying (see for review Allison & Bussey, 2016). Various studies find that bystanders are more likely to intervene when witnessing immoral online behaviour when they consider themselves as capable (Gini, Albiero, Benelli, & Altoè, 2008; Hay & Meldrum, 2010; Thornberg & Jungert, 2014). Relationships between self‐evaluative traits and responses to being provoked online are unknown, although both sense of coherence and self‐esteem are related to social functioning, as detailed next.

SOC is related to emotion regulation, social perception, and social skills (for an overview, see Eriksson & Lindström, 2005). These findings consistently indicate that individuals with a high level of SOC are less likely to perceive challenging situations as threatening and unmanageable (e.g., Feldt, Kokko, Kinnunen, & Pulkkinen, 2005). Consequently, high levels of SOC are associated with lower levels of negative affect and with fewer aggressive, hostile, and disruptive behaviours among both adolescents and adults (e.g., Cederblad, Ruksachatkunakorn, Boripunkul, Intraprasert, & Höök, 2003). Overall, the findings indicate that higher levels of SOC are related to higher levels of (perceived) social competence (e.g., Cederblad et al., 2003).

With respect to self‐esteem, findings of a recent longitudinal study suggest that adolescents' self‐esteem predicts a firm belief in being capable of effectively dealing with negative emotions (Caprara, Alessandri, Barbaranelli, & Vecchione, 2013). Indeed, prior work shows that adolescents' level of self‐esteem is related to less negative affect and fewer aggressive and antisocial behaviours, but instead to more anger control after a negative event (e.g., Donnellan, Trzesniewski, Robins, Moffitt, & Caspi, 2005). Flanagan, Hoek, Ranter, and Reich (2012) show that even in a situation in which adolescents are bullied and victimized, adolescents' level of self‐esteem is related to forgiving. In line with these findings, it is likely that higher levels of self‐esteem will be related to fewer responses that can exacerbate the peer‐conflict situation.

Yet despite these seemingly consistent findings, caution must be taken as other studies indicate a dark side of having a positive self‐view. That is, inflated and unstable beliefs about one's competence can lead to easily feeling threatened. In these cases, self‐esteem is related to more aggressive and defensive behaviour in challenging (social) situations (Baumeister, Smart, & Boden, 1996). Moreover, as detailed next, adolescents' demographic variables such as gender and ethnicity may play a moderating role in how adolescents' traits influence their expressions of displeasure in online peer‐provoking situations.

### 1.3 Gender and ethnicity

Developmental scholars widely agree that boys and girls are socialized differently, and that as a consequence gender differences exist in how children and adolescents respond in peer‐conflict situations (e.g., Connolly et al., 2015; Underwood, Hurley, Johanson, & Mosley, 1999). Generally, studies show that girls are less likely to be confrontational and aggressive, but instead express their displeasure more indirectly than boys in order to maintain harmony and prevent the relationship from being damaged (Keener, Strough, & DiDonato, 2012; Leary & Katz, 2005). At the same time, research in adults suggests that self‐evaluative traits moderate these gender differences. For example, Nunn and Thomas (1999) show that when angered, men with low levels of self‐esteem lash out by administering the loudest blasts of white noise to the confederate, whereas women with low levels of self‐esteem control themselves by administering the softest blasts.

As these studies underline the importance of taking gender into account when studying adolescents' responses to peer provocations, diversity research stresses the need to do the same with respect to ethnicity (Plaut, 2010). With respect to peer provocations, differences between adolescents from majority and minority groups are plausible. That is, minority group members who have experience with discrimination or racism may interpret and consequently respond to peer provocations differently than majority group members. In the Netherlands and also in other Western European countries, attention of the general public is drawn to a particular group of minority adolescents: adolescents of Moroccan descent. In the Dutch media, this minority adolescent group is often portrayed as having behavioural problems, including being quick to anger and responding aggressively. Consequently, Moroccan‐Dutch individuals report high levels of perceived discrimination (Schrier et al., 2014). Although studies show that parents and teachers report more externalizing problems among Moroccan‐Dutch than Dutch adolescents (Stevens et al., 2003), these differences do not appear in self‐reports. In one set of studies, Dutch and Moroccan‐Dutch adolescents' self‐reported anger communication was examined (Novin, Banerjee et al., 2012; Novin & Rieffe, 2012). The only significant group difference found was that not Moroccan‐Dutch, but Dutch adolescents reported more aggressive responses in response to hypothetical anger‐eliciting social situations. These inconsistencies in findings might be related to different informants, and an experimental set‐up as in the present study is likely to help clarify the actual differences in responses to peer provocations between Dutch and Moroccan‐Dutch adolescents.

### 1.4 The present study

The aims of the present study were to examine adolescents' expressions of displeasure before and during online peer provocation and the extent to which SOC, self‐esteem, gender, and ethnicity contribute to these expressions. We focused on adolescents because they report spending a considerable time on a daily basis communicating with others on Internet platforms (Livingstone et al., 2014) and because adolescence is an important period in which self‐evaluations are created and are salient in their lives (Harter, 2012). Going beyond self‐report measures of how adolescents respond to (online) peer provocations, we developed an ecologically valid online computerized game in which the peer teammate acted provokingly. In light of validating this game, we analysed participants' happiness and anger before and after the game, expecting that the game would increase anger and decrease happiness. Crucially, we also analysed the comments that the adolescents wrote to the fictitious peer. Based on prior findings as described above, we expected that feeling confident about oneself (self‐esteem) and about the manageability of the world (sense of coherence) would be related to fewer responses in which displeasure is expressed. Yet these effects could be moderated by gender (Nunn & Thomas, 1999), such that especially boys with low levels of SOC and self‐esteem are more likely to express displeasure. We also tested for possible moderating effects of ethnicity but did not have specific expectations about these effects.

## 2 METHOD

### 2.1 Participants and procedure

Ninety‐two Moroccan‐Dutch (47 boys; *M*_age_ = 15.7; *SD* = 8 months) and 75 Dutch (43 boys; *M*_age_ = 15.6; *SD* = 7 months) adolescents were individually tested in a quiet room in school during or after school hours.^1^ All Moroccan‐Dutch adolescents were born in the Netherlands or moved to the Netherlands before their tenth birthday. All Dutch adolescents and both their parents were born in the Netherlands.^2^ Prior to the study, institutional review board approval and participants' consent were obtained. At the end of the study participants were thanked and thoroughly debriefed. Fifty Euro was raffled among all participants.

### 2.2 Measures

#### 2.2.1 Peer‐provoking game

A computerized game was developed to examine adolescents' chat responses in peer‐provoking situations. Participants were randomly assigned to the condition of playing in a team with a Dutch or a Moroccan‐Dutch peer (matched for gender). Computerized instructions informed participants that they would play an online “cooperation” game with an unknown peer from another school in a different city. On the left side of the screen moneybags were popping on and off the screen. In five alternating rounds (including one practice trial that the fictitious peer started; blue = participant; red = fictitious peer), the task was to catch as many moneybags as quickly as possible as a team and drag them into the treasure chest. The participant was able to follow the “real‐time” actions of the fictitious team player on screen. The team with the most moneybags would win 50 Euro.

On the right side of the screen, the name and a photograph of the fictitious peer was displayed. In turn, we took a fake picture of the participant at the start of the game, which we supposedly uploaded. Underneath the picture there was a chat box, which allowed the players to communicate when it was not their turn. In total, participants could provide written responses after each round (1–4) and a final message at the end of the game. The preprogrammed chat messages from the fictitious peer built up from rather aggravating (e.g., “Hey, I really need that money!” and “You can do better”) in the first round to real provocation in the fourth round (e.g., “You're as slow as a turtle” and “It's really annoying you ruined the game”). For ethical reasons, provoking comments were not personal but merely related to game playing. Two to three preprogrammed comments from the fictitious peer were shown during each round. Typos and typical chat terms were included in the fictitious peer comments to make it look real. All chat responses stayed on the screen during the entire game. In addition to the provoking chat messages, performance of the fictitious peer steeply declined from satisfactory to deliberately obstructing the game (i.e., moving the moneybags to different parts of the screen rather than to the treasure chest).

#### 2.2.2 Mood ratings

Our manipulation check testing whether the game increased feelings of anger and decreased feelings of happiness consisted of two mood questions, which were presented twice. Participants rated their level of anger and happiness before and after the game on a 5‐point scale ranging from 0 ‘*not at all’* to 4 ‘*very much’*.

#### 2.2.3 Sense of coherence

The Dutch sense of coherence scale (Jellesma, Terwogt, & Rieffe, 2006) consists of 13 items, asking participants respond to statements on a 5‐point scale from 1 “*almost never*” to 5 “*almost always*” (α = .82). An example of an item is “How often do you have the feeling that you are in an unfamiliar situation and don't know what to do?” Two items that are recoded have a different response format from 1 “*like it a lot*” to 5 “*don't like it at all*.”

#### 2.2.4 Self‐esteem

The Dutch Rosenberg's self‐esteem scale (Van der Linden, Dijkman, & Roeders, 1983) consists of 10 items that are rated on a 4‐point scale ranging from 0 “*strongly disagree*” to 3 “*strongly agree*” (α = .86). Examples are “Generally I am content about myself” and “Sometimes I think I am not good in anything.”

### 2.3 Coding of the responses

Participants' responses during each round and the final comment were coded on the basis of presence (1) or absence (0) of expressions of displeasure^3^ (potentially aggravating comments in which one's displeasure, frustration, or anger towards the game or the other player is expressed, e.g., “this is a stupid game” or “haha, now you don't get your money”).^4^ There was no peer provocation in the first round. In this baseline round, the number of expressions of displeasure was 0 or 1. In each of the remaining three rounds and the final message (four rounds in total), which was during or after the peer‐provocation, the number of expressions of displeasure was also 0 or 1, making the summed number ranging from zero (no such response) to 4. All responses were coded by two independent judges, which revealed Cohen's kappa varying from .80 (Rounds 2 and 3) to .95 (Round 1). Disagreements were resolved through discussion.

### 2.4 Analyses plan

First, the effect of the peer‐provoking game on self‐reported happiness and anger was tested by a Gender × Ethnic Group (Dutch, Moroccan‐Dutch) × Time (before versus after) × Emotion (anger versus happiness) anova. Second, we tested whether there were initial group differences in terms of gender and ethnicity in adolescents' baseline responses when there was no peer provocation using chi‐square test. Next, a Gender × Ethnic Group anova was conducted to test the chat responses during and after peer provocation. Here, the dependent variable was the proportion of expressing displeasure (i.e., number of chat responses expressing displeasure divided by total number of chat responses). We were not able to test whether the number of no responses during and after peer provocation differed as a function of gender and ethnicity, given the low number in each cell (*n* < 5).

Finally, we conducted a multiple regression analysis with the proportion scores expressing displeasure during chats as the dependent variable. In the first step, Gender (0 = Girl and 1 = Boy), Ethnic Group (0 = Moroccan‐Dutch and 1 = Dutch), centred SOC, and centred Self‐esteem were added. In the second step the two‐way interactions between demographic variables, Gender, Ethnic Group with SOC, and Self‐esteem were added. In the final step, we added the three‐way interactions between these independent variables (i.e., Gender × Ethnic Group × Self‐esteem and Gender × Ethnic Group × SOC). We used bootstrapping procedures (resampled 1,000 times and used the percentile method to create 95% confidence intervals).

## 3 RESULTS

### 3.1 Manipulation check

Our anger‐evoking task was successful in inducing negative affect. A main effect of Emotion was revealed, *F*(1, 159) = 118.95, *p* < .001, *d* = 1.73, which was qualified by an Emotion × Time interaction, *F*(1, 159) = 47.84, *p* < .001, *d* = 1.10. As expected, adolescents reported a decrease in happiness (from *M* = 1.79, *SD* = 0.99 to *M* = 1.28, *SD* = 1.31) and an increase in anger (from *M* = 0.17, *SD* = 0.60 to *M* = 0.71, *SD* = 1.12). No effects of gender or ethnic group were found (all *Fs* < 2.79, *ps* > .10).

### 3.2 Adolescents' chat responses

As can be seen in Table 1, during the baseline round, before participants were provoked, 25% of the participants did not provide a written response. Furthermore, only a minority of participants (12%) expressed their displeasure during this baseline round. These percentages did not differ as a function of gender and ethnicity (all *ps* > .06). During and after peer provocation, expressions of displeasure were much more common (79% of the participants expressed their displeasure at least once). The mixed anova with the proportion of responses expressing displeasure during and after peer provocation as dependent variable showed no significant main nor interaction effects of Gender and Ethnic Group (all *Fs* < 1.45, *ps* > .23).

**Table 1:** Percentages and mean scores (standard deviations) of no responses, expressions of displeasure, self‐esteem, and sense of coherence as a function of time and ethnic group

	Dutch		Moroccan‐Dutch	
	Male	Female	Male	Female
Baseline				
No responses	20.9%	15.6%	31.9%	28.9%
Expressions of displeasure	16.3% (37.4%)	3.1% (17.7%)	17.0% (38.0%)	8.9% (28.8%)
During and after peer provocation				
No responses	2.3%	6.3%	2.1%	2.2%
Proportion expressing displeasure	46.8% (32.2%)	46.8% (33.1%)	50.1% (33.0%)	38.0% (31.1%)
Sense of coherence	3.54 (0.45)	3.28 (0.50)	3.54 (0.62)	3.75 (0.57)
Self‐esteem	2.31 (0.49)	1.78 (0.48)	2.37 (0.48)	2.22 (0.49)

### 3.3 Factors influencing adolescents' expressions of displeasure

Table 2 displays the results of the multiple regression analysis, revealing a main effect of SOC, indicating that higher levels of SOC were associated with expressing displeasure less often. Furthermore, an interaction effect between self‐esteem and ethnic group was revealed. Higher levels of self‐esteem were associated with relatively more responses displaying displeasure in Moroccan‐Dutch adolescents (B = 0.22, 95% CI [.09, .36] but not in Dutch adolescents (B = −0.07, 95% CI [−.22, .08]). The three way interactions were not significant.

**Table 2:** Multiple regression analyses (enter method) gender, ethnic group, sense of coherence, and self‐esteem on proportion scores of expressions of displeasure during and after peer provocation

	Expressing displeasure	
	R^2^_adj_ a	B [95% CI]
Step 2	.06*	
Gender		0.04 [−0.07, 0.15]
Ethnic group		0.02 [−0.91, 0.13]
Self‐esteem (SE)		0.16 [−0.01, 0.32]
Sense of coherence (SOC)		−0.19 [−0.35, −0.04]
Ethnic group × SE		−0.29 [−0.49, −0.08]
Ethnic group × SOC		0.23 [−0.01, 0.45]
Gender × SE		0.11 [−0.09, 0.32]
Gender × SOC		0.11 [−0.09, 0.32]

*Note*. Unstandardized beta‐coefficients and 95% confidence intervals (CI) are reported for the significant regression model only. Note that if zero is not in the 95% CI, the predictor significantly contributes to the regression model.

a Adjusted R^2^_adj_ for Step 1: −.01 and for Step 3: .06.

* *p* < .05.

## 4 DISCUSSION

In the digital world where youngsters spend much time online and can experience provocation just as in the “real world”, it is crucial to gain insight into what is happening during online peer interactions. Given that responses in self‐report studies do not necessarily reflect adolescents' actual responses in peer‐provoking situations, we developed an online computerized game in which the peer teammate from another school in a different city acted provokingly. The participant had never met this other peer and would probably never meet him or her, but a picture of the peer was on display during the game. Unbeknownst to the participant, the game was actually offline, rigged, and there was no actual teammate. We asked participants how they felt before and after the game and content coded their chat responses.

Our findings show that the game successfully elicited frustration; self‐reported happiness levels decreased, and self‐reported anger levels increased. Moreover, expressions of displeasure were much more common after adolescents were provoked by a peer than before. When adolescents did not express their displeasure, they either did not provide a written response or responded in a potentially neutralizing manner in which no aggravation was expressed. We did not find any gender or ethnic group differences in their self‐reported mood and responses. Regarding ethnicity, this supports earlier studies using hypothetical peer‐conflict situations (Novin, Banerjee et al., 2012; Novin & Rieffe, 2012). As expected, we did find that adolescents' self‐evaluations (i.e., level of SOC and level of self‐esteem) mattered for how adolescents responded to the peer‐provoking situation, and that some of these effects were moderated by ethnicity.

We predicted that adolescents' perception about one's abilities to cope with challenging (social) situations would be associated with fewer expressions of displeasure. That is, prior work indicates that people who have higher levels of SOC are less likely to perceive stressful situations as threatening (Feldt et al., 2005) and are less likely to behave in an aggressive, hostile, or disruptive manner (e.g., Cederblad et al., 2003). In a similar vein, we found that higher levels of SOC predicted fewer expressions of displeasure that could potentially escalate the conflict.

We expected a similar pattern with respect to self‐esteem: People who feel confident about their capacities and abilities perceive interpersonal conflict situations as less threatening and have less need to show their displeasure (e.g., Donnellan et al., 2005). However, our findings paint a different picture. We found that higher levels of self‐esteem were related to more expressions of displeasure, but only in the Moroccan‐Dutch group. Prior work indeed indicates that an inflated and unstable self‐esteem is related to more aggressive and defensive behaviour in situations in which one feels threatened (e.g., Baumeister et al., 1996). Yet, given the low number of actual aggressive responses in our sample (6.3% of the total number of responses and no responses), it seems more plausible that feeling confident about oneself was associated with feeling confident and competent about expressing one's displeasure, thus with openly saying what is on one's mind, as a response to peer provocation.

It is remarkable that self‐esteem was only related in the Moroccan‐Dutch group and not the Dutch group. Although we can only speculate about possible explanations regarding these ethnic group differences, existing theory can provide a guideline for interpretation. According to social identity theory (Tajfel & Turner, 1986), a part of one's self‐concept is derived from the social groups one identifies with. This may be especially be true for ethnic minority adolescents, such as Moroccan‐Dutch adolescents, for whom ethnic identity formation is part of their adolescent lives (Verkuyten & Fleischmann, 2017). Consequently, it might be that self‐esteem is more likely to influence the behaviour of ethnic minority adolescents than those from a majority group (Wissink, Dekovic, Yagmur, Stams, & de Haan, 2008). Somewhat related to this, it is possible that in light of their minority status and perceiving high levels of discrimination (Schrier et al., 2014), Moroccan‐Dutch adolescents perceive (online) peer provocations as more severe and threatening than their Dutch peers. These differences in appraisals might explain how self‐esteem is differentially related to responses in the two ethnic groups. Future studies are needed to test whether our findings can be replicated and are generalizable to other situations (e.g., provocations by a friend).

Our findings are based on our newly developed, ecologically valid online provocation game. Going beyond self‐reports that have crucial disadvantages (e.g., being subject to response bias such as social desirability and responses being semantic rather than episodic), our game provides insight into adolescents' actual expressions and communication styles when being (mildly) provoked online. This methodology opens the door to addressing other relevant research questions. First, future research could manipulate the extent of online provocation and examine emotional and cognitive processes using for example social information processing models (e.g., Crick & Dodge, 1994). This will shed light on when adolescents interpret provocations as benign and when they interpret them as more serious, providing explanations for the subsequent responses.

Second, the current online peer interaction was between the participant and an unknown peer. Yet in social network platforms, there are often multiple others (friends and strangers) virtually present; their presence may influence the participant's moral judgments and decisions. In future work, it might be useful to distinguish between provoking situations with or without virtual onlookers. Third, it would be insightful to examine the extent to which the newly developed provocation game elicited frustration not only based on differences in self‐reported emotions but also based on physiological measures such as heart rate and skin conductance. This would help to further validate the instrument.

Fourth, we focused on two indices of perceived self‐evaluation in relation to adolescents' responses. Other factors contributing to how youngsters respond in online peer‐provoking situations might be adolescents' perceived social support, problem solving–coping skills, and levels of empathy. These, among other factors, have been found to serve as protective factors in more severe peer‐conflict situations, such as adolescent bullying (e.g., Fanti, Demetriou, & Hawa, 2012).

Although online platforms can feel like a black box to parents where children and adolescents are potential victims of peer provocations, we show that adolescents show their displeasure about half the time when being (mildly) provoked online. Especially for the minority group (i.e. Moroccan‐Dutch), self‐evaluative traits were related to online chat expressions when being provoked by a peer. Understanding both risk and protective factors that contribute to adolescents' responses to an online interpersonal conflict situation is imperative in the digital world we live in and will contribute to effective prevention and intervention.

## ENDNOTES

1 The study was part of a larger study examining emotional functioning of bicultural adolescents (Novin, 2011).

2 The sample originally consisted of 186 adolescents, but 12 participants were excluded because they either did not fall into the Moroccan‐Dutch or Dutch group (*n* = 4), or because they had noticed that the computerized reactions were pre‐programmed (*n* = 8).

3 Responses that do not fall into this category were potentially neutralizing comments in which no aggravation is expressed by reacting positively, talking neutrally about the game, or about one's own performance, for example, “come on, you can do it” or “this game is fun” or “are you Moroccan?” or “I'm not good at the game.”

4 Other displeasure expression examples include “I'm not a turtle” or “Be nice” or “You're not playing so good yourself” or “WTF be real” or “you're supposed to encourage me.” The displeasure category included aggressive responses, including swearing or insulting comments. The number of these responses was too low (6.3% of total number of responses and no responses) to examine as a separate category. Results did not differ whether these aggressive responses were included in or excluded from the displeasure category.
